# Wound formation in patients with Rutherford category IV disease after endovascular therapy: rates and risk factors

**DOI:** 10.1186/s42155-024-00500-3

**Published:** 2024-12-07

**Authors:** Toshihiko Kishida, Shinsuke Mori, Kohei Yamaguchi, Masakazu Tsutsumi, Norihiro Kobayashi, Yoshiaki Ito

**Affiliations:** Department of Cardiology, Saiseikai Yokohama City Eastern Hospital, Yokohama, Japan

**Keywords:** Rutherford category IV disease, Endovascular therapy, Wound formation, Risk factors

## Abstract

**Background:**

Lower limb peripheral artery disease classified as Rutherford category IV, is characterized by lower limb ischemic pain both during exertion and at rest. This disease has an unclear course. We aimed to evaluate outcome predictors in this patient group after endovascular therapy. This single-center, retrospective, observational study included 234 consecutive patients (264 limbs), between April 2007 and December 2020. We investigated the disease clinical course after endovascular therapy. The primary endpoint was the wound formation rate 3 years after endovascular therapy.

**Results:**

The mean observation period was 48.2 ± 8.9 months. The patients (61.9% male; mean age, 76 ± 10 years) presented with diabetes (64.1%), and received hemodialysis with chronic kidney disease (35.0%) and ambulatory treatment (85.0%). The average ankle-brachial index before endovascular therapy was 0.69 ± 0.23. Skin perfusion pressure on the dorsal and plantar sides was 38 ± 13 mmHg and 36 ± 12 mmHg, respectively. The wound incidence rates at 1, 2, and 3 years after endovascular therapy were 8.3%, 11.4%, and 14.4%, respectively. Multivariate analysis revealed the following factors associated with wound formation: P2 in inframalleolar/pedal disease category in the Global Limb Anatomical Staging System (hazard ratio: 1.73, 95% confidence interval: 1.22–2.83, *P* = 0.01), non-ambulatory status (hazard ratio: 1.09, 95% confidence interval: 1.11–1.36, *P* = 0.02), intervention up to infrapopliteal lesion (hazard ratio: 1.55, 95% confidence interval: 1.17–2.46, *P* = 0.03), and patient with chronic kidney disease on hemodialysis (hazard ratio: 1.61, 95% confidence interval: 1.32–2.18, *P* = 0.03).

**Conclusions:**

The 3-year incidence of wound onset in this study was 14.4%. Factors associated with this outcome included P2 in the Global Limb Anatomical Staging System, non-ambulatory status, intervention up to infrapopliteal lesion, and patient with chronic kidney disease on hemodialysis.

## Background

Lower limb peripheral arterial disease (PAD) is a condition that involves peripheral blood flow reduction. The causes include arteriosclerosis (e.g., due to diabetes or dialysis) [1], non-atherosclerotic disease caused by vasculitis or abnormal vascular development [2], and functional disease with no abnormality in the arterial wall structure [3]. In Japan, the prevalence of lower limb PAD is 1.1% [4] among those without risk factors such as smoking, hypertension, diabetes, dyslipidemia, and cerebrovascular disease [5]. On the other hand, the risk increases 4 to 20 times in patients with some atherosclerosis-related factors [6–8]. Ischemic symptoms lower limb PAD progress with a history of arteriosclerosis, and what starts as intermittent claudication may develop into wound development, becoming infected and life-threatening, thus requiring amputation.

Treatment for patients with lower limb PAD Rutherford category IV disease generally includes both medical management and revascularization. Antiplatelet drugs may also improve symptoms and prevent cardiovascular events [9]. If patients present with ischemic rest pain, revascularization (endovascular therapy [EVT] or surgical reconstruction) may be considered; the choice of method should be determined based on the lesion site and length and any risks of surgical complications [9]. EVT is often selected as a revascularization method because of its low invasiveness and satisfactory safety profile [10]. Tsuchiya et al. [11] previously reported that surgical reconstructions are rarely considered in this patient group. However, some patients present with wound formation after EVT.

Among lower limb PAD, Rutherford category V or VI is a determining factor for life prognosis. Some studies have suggested increased rates of cerebrovascular and cardiovascular events [12], with an estimated 1-year survival rate without revascularization at 22% [13]. Thus, Rutherford category IV patients must receive appropriate treatment and examination of lower extremity to avoid wound formation. However, the risk factors associated with wound formation after EVT are unclear and there have been no reports of Rutherford category IV disease. Therefore, in this study, we aimed to examine these factors.

## Methods

### Study design and patient enrollment

**Fig. 1:**
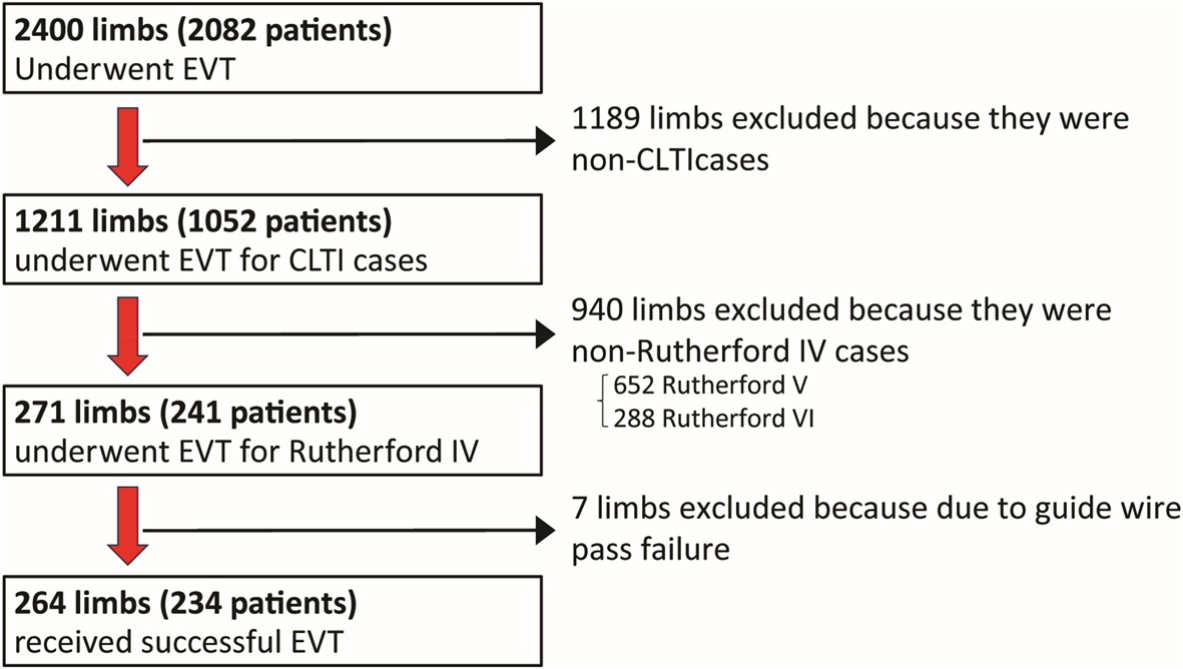
Study flowchart

This was a single-center, retrospective, observational study. We enrolled 234 patients (264 limbs) who underwent EVT to improve ischemic rest pain between April 2007 and December 2020 at our hospital. EVT was indicated for patients with rest pain that did not abate with drug. This study included patients whose EVT was successful. The study flowchart is presented in Fig. 1.

Before EVT, all patients included in this study were started on treatment for untreated risk factors for atherosclerosis, such as hypertension, diabetes, and dyslipidemia. Management of these factors continued during follow-up at our hospital or another hospital.

After discharge, patients were followed up during outpatient examinations performed at intervals of 3–6 months, which included interviews and physical examinations, and ankle-brachial index (ABI) and ultrasound examinations at least once per year. Restenosis or reocclusion of treated lesions was assessed by ultrasound or contrast examination. In cases of wound formation after EVT, the patients were monitored in an outpatient setting by specialists including dermatologists, plastic surgeons, and orthopedic surgeons. The primary endpoint was the 3-year wound formation rate after EVT. The secondary endpoints were the 3-year major amputation-free survival and mortality rates after EVT. We also performed univariate and multivariate analyses to identify predictors of wound formation after EVT.

Univariate analysis included factors that have been reported to be likely to progress to chronic limb-threatening ischemia (CLTI) or worsen the prognosis: older age [14], female gender [15], atherosclerotic factors (hypertension [5], diabetes [5], dyslipidemia [16], patient with chronic kidney disease [CKD] on hemodialysis [17]), frailty (low body weight [14], hypoalbuminemia [14], high inflammatory response [14], decreased ADL [18]), lesions that are difficult to revascularize [19], and related medications such as cilostazol [20] and statin [21].

Variables with a probability (p)-value of < 0.05 in the univariate analysis were included in the multivariate analysis.

Data on clinical, perioperative, and demographic characteristics were extracted from medical records. This study complied with the Declaration of Helsinki and was approved by the hospital ethics committee.

We posted information about this study on our hospital website and gave participants the opportunity to opt out; those who did not were considered to have provided tacit consent for study participation.

### Definitions

Patients with Rutherford category IV disease were defined as those presenting with cold and rest pain in the lower extremities and were diagnosed with lower limb ischemia based on imaging test findings, including computed tomography or magnetic resonance angiography; physiological tests such as ABI, or skin perfusion pressure (SPP), or ultrasound examination; and lower limb angiography.

For EVT, procedural success was defined as adequate blood flow to the toes, confirmed by contrast imaging studies. The presence of wound formation was determined with a joint examination by a plastic surgeon and a cardiologist. We used the Wound, Ischemia, and Foot Infection (WIfI) classification [22] to assess wound severity.

To evaluate blood flow in the inframalleolar (IM) region after EVT, we used the IM/pedal disease descriptor in the Global Limb Anatomic Staging System (GLASS-IM) [23]. This system classifies blood flow into three stages: P0, any arteries crossing the ankle into the foot with an intact pedal arch; P1, any artery crossing the ankle into the foot with or without a severely diseased pedal arch; and P2, no arteries crossing the ankle into the foot. Restenosis was defined as > 50% stenosis on computed tomography or contrast examination or > 2.4 of peak systolic velocity ratio on ultrasound examination.

### Statistical analysis

Continuous variables with normal distributions are presented as the means ± standard deviations. Categorical variables are presented as frequencies (percentages). Wound formation rates were estimated using the Kaplan–Meier method. Independent outcome predictors were identified using univariate and multivariate Cox proportional hazard regression analysis; multivariate models included variables that were statistically significant in univariate models. *P*-values < 0.05 were considered statistically significant. All statistical analyses were performed using R version 3.4.1 (The R Foundation for Statistical Computing, Vienna, Austria).

## Results

The observation period was 48.2 ± 8.9 months. Patient baseline characteristics are shown in Table 1. The mean age was 76 ± 10 years and 61.9% of them were male. The presenting comorbidities were diabetes (64.1%) and patient with CKD on hemodialysis (35.0%). In addition, 85.0%, 9.9%, and 5.1% of the patients had ambulatory status, were wheelchair users, and were bedridden, respectively.

**Table 1:** Participant baseline characteristics (*N* = 234)

Variable	
Age (y)	76 ± 10
Male sex, n (%)	145 (61.9)
Body mass index, mean ± SD (kg/m [2])	21.2 ± 2.2
Previous history of lower extremity trauma, n (%)	5 (2)
Hypertension, n (%)	205 (87.6)
Diabetes mellitus, n (%)	150 (64.1)
Dyslipidemia, n (%)	88 (37.6)
Smoker, n (%)	61 (26.1)
Chronic kidney disease, n (%)	184 (78.6)
Patient with CKD on hemodialysis, n (%)	82 (35.0)
Coronary artery disease, n (%)	126 (53.8)
Cerebral vascular disease, n (%)	42 (17.9)
Ejection fraction < 40%, n (%)	30 (12.8)
Activities of daily living	
Ambulatory, n (%)	199 (85.0)
Wheelchair user, n (%)	23 (9.9)
Bedridden, n (%)	12 (5.1)
Laboratory data	
Hemoglobin, g/dL	11.2 ± 1.9
Albumin, g/dL	3.7 ± 0.6
CRP, mg/dL	1.0 ± 2.3
Hemoglobin A1c, %	6.0 ± 1.3
Medication	
Cilostazol, n (%)	96 (41.0)
Statin, n (%)	63 (26.9)

Continuous data are presented as mean ± standard deviation and categoric data as number (%)

Table 2 shows lesion and procedure characteristics. The mean pre-ABI value was 0.69 ± 0.23, and pre-SPP was 38 ± 13 mmHg on the dorsal side and 36 ± 12 mmHg on the planter side. The most distal part of EVT was iliac (14.7%), femoropopliteal (FP) (43.9%), and infrapopliteal (IP) (41.4%). 37 limbs were classified as the femoropopliteal disease descriptor in the Global Limb Anatomic Staging System (GLASS-FP) [23] grade 3 or 4, and 62 limbs were classified as the infrapopliteal disease descriptor in the Global Limb Anatomic Staging System (GLASS-IP) [23] grade 3 or 4.

**Table 2:** Lesion and procedure characteristics (*N* = 264)

Before EVT	
Pre-ABI	0.69 ± 0.23
Pre-SPP dorsal, mean ± SD (mmHg)	38 ± 13
Pre-SPP planter, mean ± SD (mmHg)	36 ± 12
Lesion location	
Only iliac region, n (%)	36 (13.6)
Only femoropopliteal region (FP), n (%)	89 (33.7)
Only infrapopliteal region (IP), n (%)	65 (24.6)
Iliac + FP, n (%)	17 (6.4)
Iliac + IP, n (%)	4 (1.5)
FP + IP, n (%)	47 (17.8)
Iliac + FP + IP, n (%)	6 (2.3)
Level of most distal intervention	
Iliac, n (%)	39 (14.7)
FP, n (%)	116 (43.9)
IP, n (%)	109 (41.4)
Iliac lesion (*n* = 63)	
Stenosis, n (%)	55 (87.3)
Total occlusion, n (%)	8 (12.7)
Severe calcification, n (%)	22 (34.9)
Lesion length, mean ± SD (mm)	54.8 ± 28.8
Reference vessel diameter, mean ± SD (mm)	7.8 ± 1.1
Used balloon diameter, mean ± SD (mm)	7.2 ± 1.2
Use of stent, n (%)	63 (100)
FP lesion (*n* = 159)	
Stenosis, n (%)	82 (51.6)
Total occlusion, n (%)	77 (48.4)
Severe calcification, n (%)	45 (28.3)
GLASS-FP grade 3 or 4, n(%)	37 (23.3)
Lesion length, mean ± SD (mm)	136 ± 81
Reference vessel diameter, mean ± SD (mm)	5.4 ± 1.0
Used balloon diameter, mean ± SD (mm)	5.1 ± 1.0
Use of drug coated balloon, n (%)	93 (58.5)
Use of stent, n (%)	49 (30.8)
IP lesion (*n* = 122)	
Stenosis, n (%)	67 (54.9)
Total occlusion, n (%)	55 (45.1)
Severe calcification, n (%)	71 (58.2)
GLASS-IP grade 3 or 4, n(%)	62 (50.8)
Lesion length, mean ± SD (mm)	105.5 ± 53.3
Reference vessel diameter, mean ± SD (mm)	2.9 ± 0.5
Used balloon diameter, mean ± SD (mm)	2.2 ± 0.5
After EVT (*n* = 264)	
Post ABI	0.90 ± 0.19
Post SPP dorsal, mean ± SD (mmHg)	56 ± 6
Post SPP planter, mean ± SD (mmHg)	60 ± 7
Run off vessel 0 or 1, n (%)	114 (43.2)
Run off vessel 2 or 3, n (%)	150 (56.8)
GLASS-IM	
P0, n (%)	95 (36.0)
P1, n (%)	127 (48.1)
P2, n (%)	42 (15.9)

Continuous data are presented as mean ± standard deviation and categoric data as number (%)

Post-EVT, the mean ABI was 0.90 ± 0.19, and SPP was 56 ± 6 mmHg on the dorsal side and 60 ± 7 mmHg on the planter side. The number of run-off vessels in the IP region before EVT was 43.2% for ≤ 1 vessels and 56.8% for ≥ 2 vessels. The GLASS-IM classification distribution was as follows: 36.0%, 48.1%, and 15.9% for P0, P1, and P2, respectively.

**Fig. 2:**
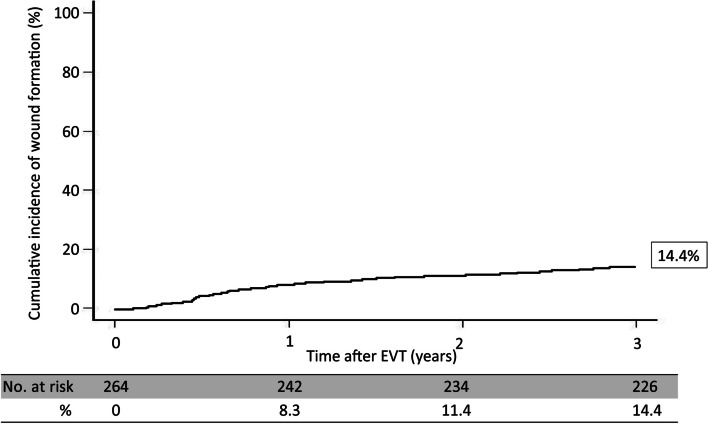
Cumulative incidence of wound formation

**Fig. 3:**
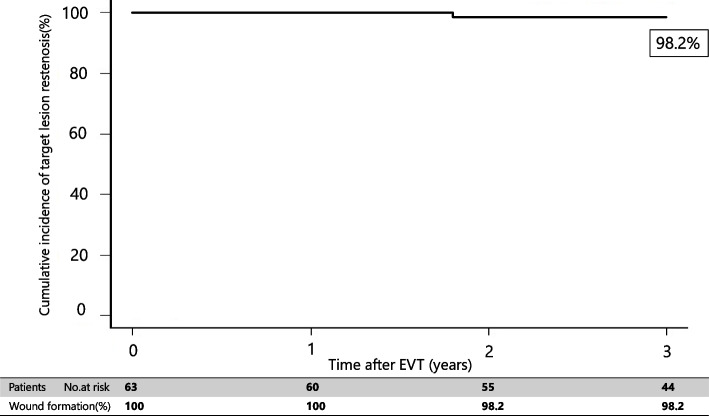
Freedom from target lesion restenosis rate of iliac lesion for 3 years after EVT

**Fig. 4:**
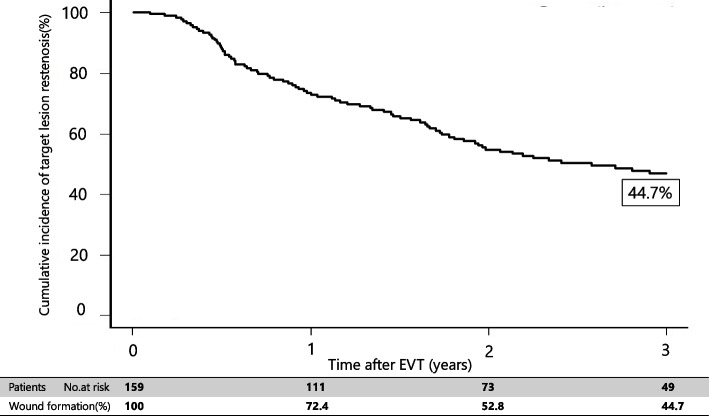
Freedom from target lesion restenosis rate of FP lesion for 3 years after EVT

**Fig. 5:**
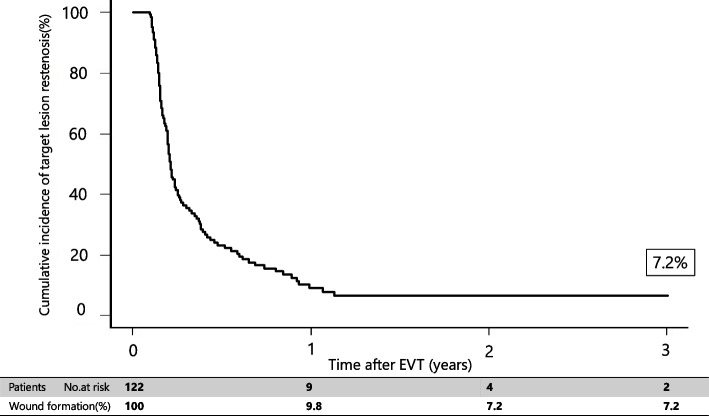
Freedom from target lesion restenosis rate of IP lesion for 3 years after EVT

The primary endpoint rates are shown in Fig. 2. The wound formation rates at 1, 2, and 3 years after EVT were 8.3%, 11.4%, and 14.4%, respectively. Tables 3 and 4 shows the site of wound formation and WIfI classification of the wound. Figs. 3, 4 and 5 shows the freedom from target lesion restenosis rate after EVT.

**Table 3:** The site of the wound formation

	Toe	Dorsum pedis	Planta pedis	Heel
Patients	22	3	4	9

**Table 4:** WIfI classification of the wounds

	Wound				Ischemic				Foot infection				Clinical stage			
	0	1	2	3	0	1	2	3	0	1	2	3	0	1	2	3
Patients	3	25	7	3	19	6	10	3	18	12	5	3	14	9	9	6

**Fig. 6:**
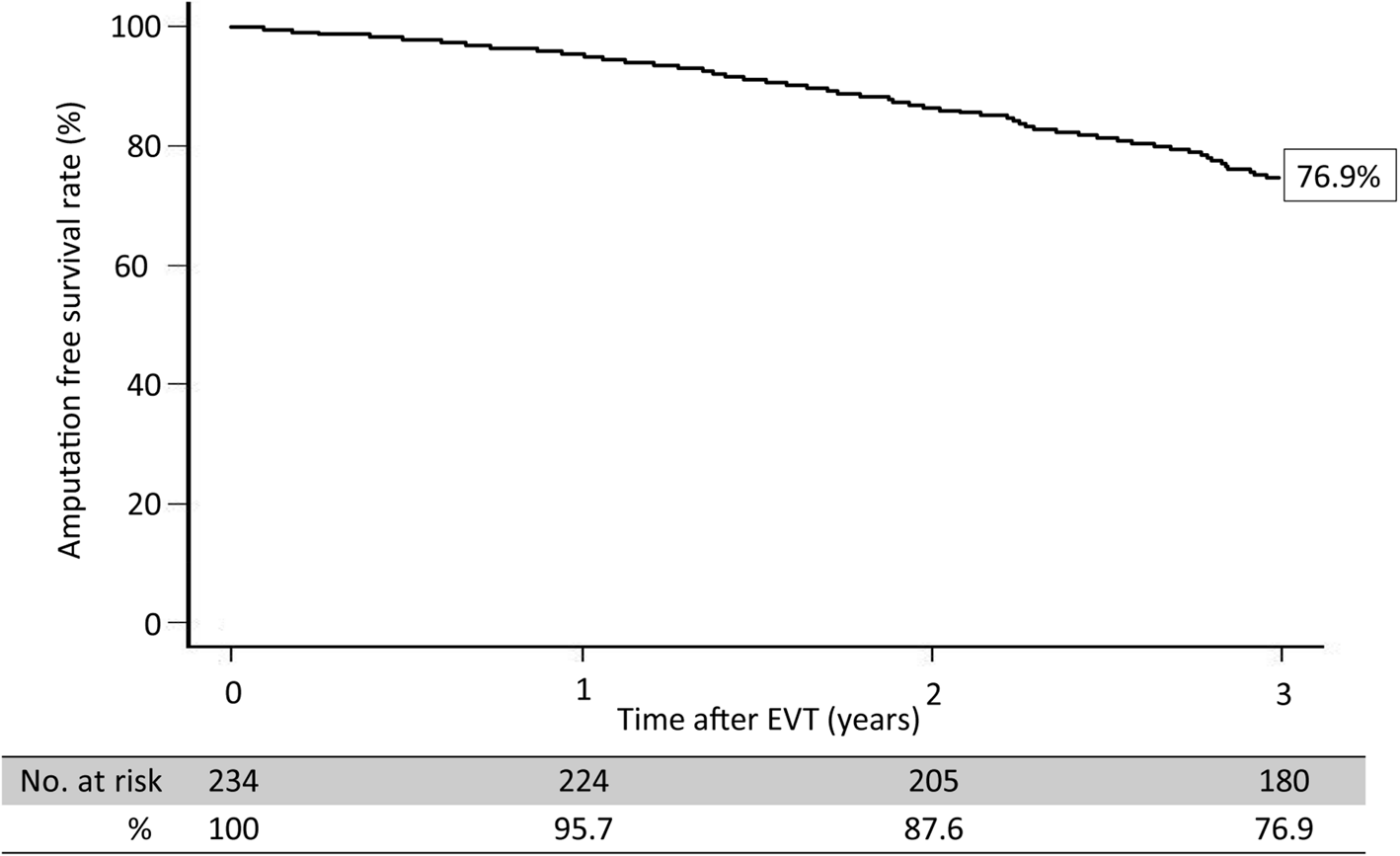
Amputation-free survival rate for 3 years after EVT

**Fig. 7:**
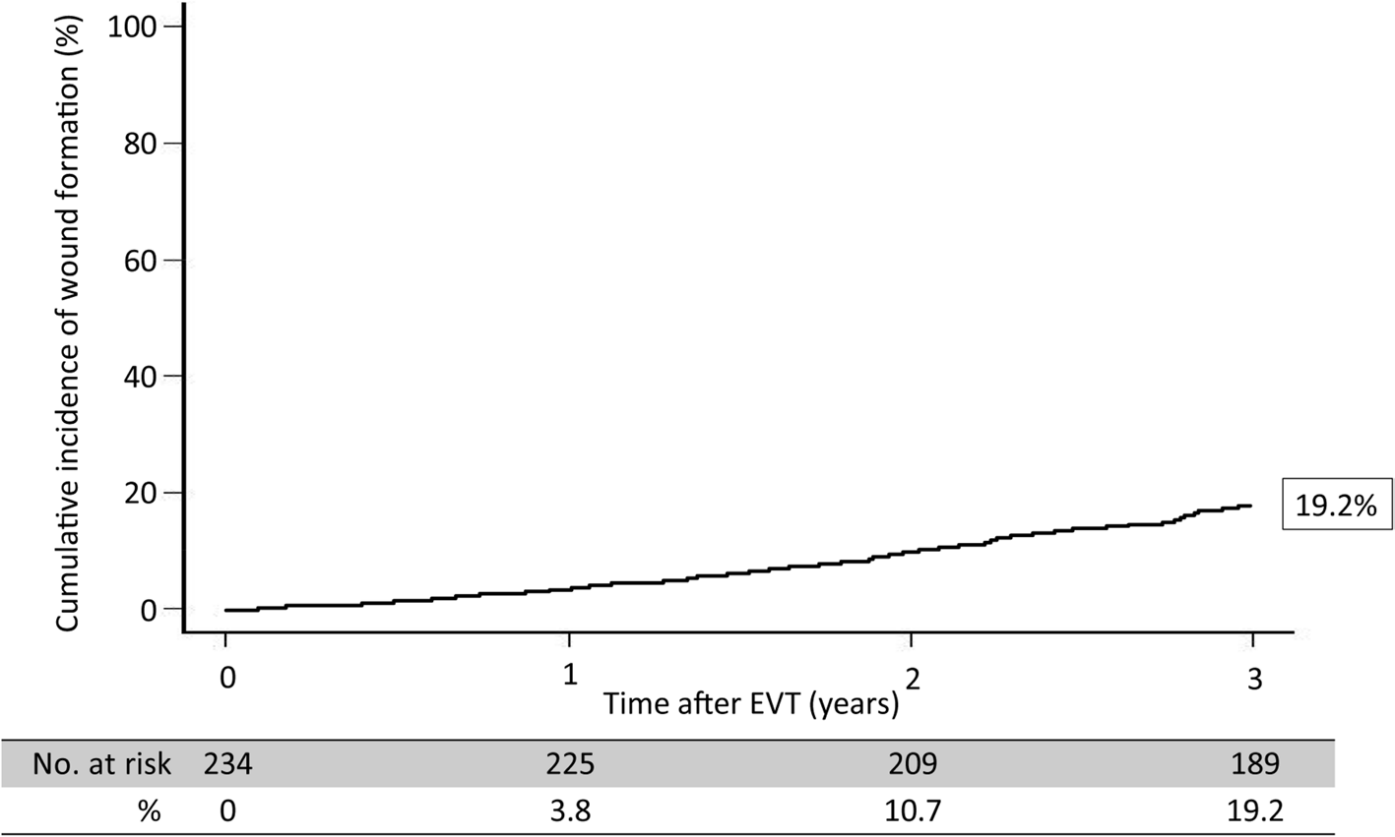
Mortality rate for 3 years after EVT

Multivariate analysis revealed that P2 in the GLASS-IM (hazard ratio [HR]: 1.73; 95% confidence interval [CI]: 1.22–2.83; *P*-value: 0.01), non-ambulatory status (HR: 1.09; 95% CI: 1.11–1.36; P-value: 0.02), intervention up to IP lesion (HR: 1.55; 95% CI: 1.17–2.46; *P*-value: 0.03), and patient with CKD on hemodialysis (HR: 1.61, 95% CI: 1.32–2.18; *P*-value: 0.03) were associated with outcomes (Table 5). In addition, the 3-year major amputation-free survival and mortality rates were 76.9% (Figs. 6 and 7) and 19.2% after EVT (Fig. 4), respectively.

**Table 5:** Risk of wound formation after EVT in wound formation using Cox univariate and multivariate analyses

	Univariate analysis			Multivariate analysis		
	HR	95% CI	*P*-value	HR	95% CI	*P*-value
Age > 85 years	1.28	0.85–1.96	0.22			
Sex (female)	0.93	0.62–1.69	0.79			
Body mass index < 18 kg/m^2^	1.08	0.78–1.58	0.65			
Ejection fraction < 40%	0.89	0.81–1.39	0.71			
Albumin < 3.0 g/dL	1.32	0.66–2.12	0.31			
CRP > 3 mg/dL	0.97	0.56–1.66	0.78			
GLASS-FP grade 4	1.13	0.75–1.67	0.55			
GLASS-IP grade 4	1.24	1.04–1.98	0.05	0.90	0.69–1.24	0.28
GLASS-IM P2	1.49	1.23–2.42	0.004	1.73	1.22–2.83	0.01
Non-ambulatory	1.62	1.22–2.45	0.01	1.09	1.11–1.36	0.02
Intervention up to IP lesion	1.72	1.23–2.32	0.009	1.55	1.17–2.46	0.03
Risk factors						
Hypertension	1.15	0.72–1.75	0.56			
Diabetes mellitus	1.34	1.16–1.82	0.05	1.26	0.77–1.69	0.22
Dyslipidemia	0.96	0.59–1.46	0.76			
Patient with CKD on hemodialysis	1.92	1.32–2.71	0.001	1.61	1.32–2.18	0.03
Medication						
Cilostazol	0.82	0.62–1.32	0.80			
Statin	0.89	0.68–1.42	0.83			

Data are based on the Rutherford classification

*HR* hazard ratio, *CI* confidence interval

## Discussion

To the best of our knowledge, this is the first study to investigate the outcome, wound development rate and predictors of patients with Rutherford category IV disease after EVT.

Several classifications have attempted to define lower limb PAD severity, with the Rutherford classification being the most commonly used [24]. Rutherford categories IV to VI are defined as CLTI and have a poorer prognosis than Rutherford categories I to III. A previous study has reported the 5-year survival rate of patients with intermittent claudication as 73.3% [25], while the 1-year mortality rate of CLTI has been estimated at 22% [13]. In addition, the 5-year amputation rate of the lower limbs has been estimated at 1–3% for patients with intermittent claudication, whereas the 6-month amputation rate in CLTI patients has been estimated at 40% [26]. In this study, the 3-year amputation-free survival and mortality rates were 76.9% and 19.2%, respectively, after EVT.

In general, 2–5% of the patients with Rutherford categories I to III disease progress to CLTI within 5 years [25]. Infection of wound is the most common cause of death in CLTI patients [27]. Patients categorized as Rutherford V or VI exhibit wounds to the lower extremities, which increase the risk of infection. In addition, revascularization by EVT is more difficult in Rutherford category V than in category IV disease because intervention on the IP artery is more frequently required [11]. Therefore, in patients with Rutherford category IV disease, it is important to ensure sufficient blood flow by reconstructing blood circulation, providing daily foot care guidance, and performing outpatient examinations to avoid wound formation.

The P2 category in the GLASS-IM indicates poor blood flow in the IM. Patients with ischemic rest pain and these risk factors require monitoring for wound formation during outpatient follow-up after EVT. Patients with poor blood flow in the IM have been shown to have an increased risk of lower limb amputation and mortality [28].

In addition, a non-ambulatory status may indicate a patient with comorbidities and poor nutrition [29], which may affect wound formation. Diabetes, heart failure, renal failure, hemodialysis, and decreased levels of albumin, total lymphocyte, and cholinesterase are closely related to the risk of wound formation [30–33], and have been included in a risk model for disease progression.

Patients with a non-ambulatory status may be prone to developing pressure sores on their heels [34]. In this study, 10 of 11 patients who developed heel wounds were non-ambulatory, suggesting that pressure ulcers are precursors to wounds.

Previous reports have indicated that among patients with CLTI, those undergoing dialysis exhibit particularly poor pedal arch patency, leading to a higher incidence of wound formation and major amputation [35]. The results of this study corroborate these findings. Patients with CLTI in Japan have different characteristics from their counterparts elsewhere, including older age, poorer nutrition, renal failure, and diabetes [36]. In the SPINCH study [17], EVT emerged as more appropriate than surgical reconstruction for patients with diabetes and renal failure; therefore, a risk assessment method that considers these characteristics is required for Japanese patients with CLTI. Moreover, there have been no reports on the incidence of wound formation after surgical reconstruction for ischemic rest pain and further research is warranted.

The limitations of this study are the relatively small sample size and its retrospective and single-center design. In addition, this study included mostly Japanese patients, and the results may differ for patients of other nationalities, as their lesion backgrounds may be different. A study with a larger sample size encompassing multiple nationalities may lead to different conclusions.

## Conclusions

In patients with Rutherford category IV disease, the 3-year rate of wound formation was 14.4% after EVT, and the associated risk factors were P2 in the GLASS-IM, non-ambulatory status, isolated IP lesion, and patient with CKD on hemodialysis. This study identified which patients in Rutherford category IV are at higher risk of developing wounds. Careful follow-up of these patients will help reduce wound development and improve outcomes after EVT.

## Data Availability

NA.

## Abbreviations

ABI: Ankle-brachial index
CI: Confidence interval
CKD: Chronic kidney disease
CLTI: Chronic limb-threatening ischemia
EVT: Endovascular therapy
FP: Femoropopliteal
GLASS-FP: Femoropopliteal disease descriptor in the Global Limb Anatomic Staging System
GLASS-IM: Inframalleolar disease descriptor in the Global Limb Anatomic Staging System
GLASS-IP: Infrapopliteal disease descriptor in the Global Limb Anatomic Staging System
IM: Inframalleolar
IP: Infrapopliteal
PAD: Peripheral arterial disease
SPP: Skin perfusion pressure
WIfI: Wound, Ischemia, and Foot Infection
